# Accuracy of transcranial color-coded duplex sonography in predicting clinical vasospasm and delayed cerebral ischemia in patients with subarachnoid hemorrhage

**DOI:** 10.1186/cc13652

**Published:** 2014-03-17

**Authors:** F Di Corte, S Pifferi, L Capuano, S Magnoni, V Conte, F Ortolano, N Stocchetti

**Affiliations:** 1 Fondazione IRCCS Ca Granda - Ospedale Maggiore Policlinico, Milan, Italy

## Introduction

Cerebral vasospasm (VSP) and delayed cerebral ischemia (DCI) play a central role in worsening the neurological outcome in patients with aneurismal subarachnoid hemorrhage (aSAH) [1]. Transcranial color-coded duplex sonography (TCCS) is a non-invasive tool to detect VSP and predict DCI, but its clinical utility is limited by low accuracy. Our aim was to perform a sensitivity/specificity analysis of TCCS in predicting clinical VSP and DCI.

## Methods

Consecutive patients admitted to our ICU for aSAH were enrolled in the study. CBF-Vm and the pulsatility index (PI) were obtained by TCCS in both mean cerebral arteries (MCAs) at two scheduled time points, <3 days (T1) and 7 to 10 days (T10), and at least once every other day after bleeding. The highest Vm between left and right and the corresponding PI were chosen for analysis. All patients underwent brain MRI plus TOF-MRI angiography at T1 and T10 to assess radiologic VSP. Symptomatic VSP was defined as a new neurologic deficit associated with radiological VSP. The occurrence of DCI was evaluated on DWI sequences. The *C *statistic (ROC curves) and nonparametric *t *test were used for analysis.

## Results

Forty-three consecutive patients were recruited. Thirty-seven patients had simultaneous TCCS measures and MRI (mean age 58 years, 28% WFNS 4 to 5). Eleven patients (30%) developed symptomatic VSP and 26 (70%) did not (NVSP). Vm increased significantly from T1 to T10 in both groups (VS: *P *= 0.03, NVSP *P *= 0.01). The accuracy of TCCS at T10 (Vm) for predicting vasospasm was described by an aUc of 0.86 (CI = 0.74 to 0.98) and a cutoff value >115 cm/seconds (90% sensitivity, 73% specificity). Interestingly, a cutoff value >168 cm/second corresponded to the best specificity (92% specificity), but low sensitivity (45%). The accuracy of PI for predicting VSP was lower (AUC 0.75). The accuracy of TCCS for predicting DCI was also poor (AUC 0.57). Considering larger DCI only (volume >2 ml), the accuracy was slightly increased (AUC 0.75).

## Conclusion

TCCS is useful to predict clinical VSP with good accuracy. The Vm cutoff value >115 cm/second might be considered as a warning threshold, while higher values (>168 cm/second) identify high-risk patients. The accuracy of TCCS in predicting DCI is low, which indicates that mechanisms other than VSP are likely to play a role.

## References

[B1] Carrera E Transcranial Doppler for predicting delayed cerebral ischemia after subarachnoid hemorrage Neurosurgery 2009 65 316 324 10.1227/01.NEU.0000349209.69973.88 19625911

